# Optogenetics and chemogenetics: key tools for modulating neural circuits in rodent models of depression

**DOI:** 10.3389/fncir.2025.1516839

**Published:** 2025-02-25

**Authors:** Shaowei Li, Jianying Zhang, Jiehui Li, Yajie Hu, Mingkuan Zhang, Haijun Wang

**Affiliations:** ^1^College of Traditional Chinese Medicine, Shandong University of Traditional Chinese Medicine, Jinan, China; ^2^The Affiliated Hospital of Shandong University of Traditional Chinese Medicine, Jinan, China; ^3^Shengli Oilfield Central Hospital, Dongying Rehabilitation Hospital, Dongying, China; ^4^College of Medical and Healthcare, Linyi Vocational College, Linyi, China

**Keywords:** optogenetics, chemogenetics, depression, neural circuits, rodent models of depression, sonogenetics, odourgenetics

## Abstract

Optogenetics and chemogenetics are emerging neuromodulation techniques that have attracted significant attention in recent years. These techniques enable the precise control of specific neuronal types and neural circuits, allowing researchers to investigate the cellular mechanisms underlying depression. The advancement in these techniques has significantly contributed to the understanding of the neural circuits involved in depression; when combined with other emerging technologies, they provide novel therapeutic targets and diagnostic tools for the clinical treatment of depression. Additionally, these techniques have provided theoretical support for the development of novel antidepressants. This review primarily focuses on the application of optogenetics and chemogenetics in several brain regions closely associated with depressive-like behaviors in rodent models, such as the ventral tegmental area, nucleus accumbens, prefrontal cortex, hippocampus, dorsal raphe nucleus, and lateral habenula and discusses the potential and challenges of optogenetics and chemogenetics in future research. Furthermore, this review discusses the potential and challenges these techniques pose for future research and describes the current state of research on sonogenetics and odourgenetics developed based on optogenetics and chemogenetics. Specifically, this study aimed to provide reliable insights and directions for future research on the role of optogenetics and chemogenetics in the neural circuits of depressive rodent models.

## 1 Introduction

Depression is a prevalent mental health disorder characterized by persistent low mood, social withdrawal, slowed thinking, cognitive impairment, insomnia, loss of appetite, and anhedonia. In severe cases, individuals may experience suicidal thoughts and behaviors (James et al., 2018). In recent years, the prevalence and incidence of depression have increased rapidly, attributed to factors such as rapid societal development, increasing societal pressure, and the global COVID-19 pandemic (Liu et al., 2020). The World Health Organization (WHO) predicts that by 2030, depression will be one of the leading causes of disability worldwide (World Health Organization, 2008). Depression has significantly impacted individuals and societies, imposing a significant economic burden on governments globally (Greenberg et al., 2021). The underlying pathogenesis of depression remains elusive, contributing to suboptimal and often recurring clinical treatment outcomes (Kessler et al., 2003).

Three primary treatment approaches are currently available for depression (Cuijpers et al., 2021). The first approach is pharmacological therapy, primarily involving selective serotonin reuptake inhibitors (SSRIs). SSRIs exert their therapeutic effects by increasing serotonergic neurotransmission in the brain, thereby improving mood. While generally well-tolerated, SSRIs may be associated with adverse events such as nausea, insomnia, and sexual dysfunction (Gartlehner et al., 2011). Selective serotonin and norepinephrine reuptake inhibitors (SNRIs) exert their therapeutic effect by increasing both serotonin and norepinephrine levels in the brain, thereby improving mood and energy. Potential adverse events associated with SNRIs may include hypertension, palpitations, and diaphoresis (Fanelli et al., 2021). Psychotherapy represents the second primary approach, with cognitive behavioral therapy (CBT) frequently employed. CBT helps individuals with depression identify and modify negative thought patterns while developing positive coping skills. Psychodynamic therapy, which focuses on unconscious conflicts and emotional patterns, aims to elucidate and address the root causes of depression, making it appropriate for patients with deep-seated needs and complex emotions. However, the efficacy of psychotherapeutic interventions warrants further investigation, and symptom relapse is a recognized concern (Lee and Cho, 2021; Leichsenring et al., 2021). Finally, transcranial magnetic stimulation (TMS) is a more recent treatment modality. TMS is a non-invasive brain stimulation technique that employs a strong magnetic field to induce an electric current in the scalp, stimulating the cerebral cortex and modulating neurotransmitter systems, neural circuits, and neuroplasticity, thus exerting an antidepressant effect. TMS has a low incidence of adverse effects, typically manifesting as mild headaches; however, symptom relapse remains a recognized concern. The emergence and development of TMS are closely associated with advances in understanding the neural circuitry underlying depression (De Risio et al., 2020).

Current first-line medications and treatment modalities for depression often exhibit limited efficacy and can cause significant side effects, underscoring the urgent need to identify the precise pathogenesis and novel therapeutic targets (Yang et al., 2018; Gorka et al., 2019; O’Reardon et al., 2007). Several studies have proposed various hypotheses that suggest a strong association between depression and factors such as monoamine neurotransmitters and their receptors, neurotrophic factors, related hormones, and specific genes (Mulinari, 2012; Lacerda-Pinheiro et al., 2014; McHenry et al., 2014; Levy et al., 2018).

The advent of techniques such as optogenetics and chemogenetics has enabled researchers to manipulate the activity of specific neuronal types in living animals precisely, shifting the focus of depression research from pathogenesis to the underlying neural circuits (Spellman and Liston, 2020). This shift has significantly advanced our understanding of depression. The combined use of optogenetics, chemogenetics, behavioral assessments, and various neurobiological assays holds immense promise for elucidating the precise pathogenesis of depression, developing novel antidepressants, and devising effective clinical treatment. Numerous neural circuits have been implicated in depression, including the dopamine-mediated mesolimbic reward pathway, the GABAergic negative-feedback inhibitory system, and the glutamatergic positive-feedback regulatory system (Su and Si, 2022; Hare and Duman, 2020; Russo and Nestler, 2013). Additionally, the roles of deeper brain nuclei, such as the dorsal raphe nucleus (DRN) and lateral habenula (LHB), are increasingly recognized in the pathogenesis of depression (Sun et al., 2022; Yang et al., 2018). This review examines the application of optogenetics and chemogenetics in studying brain regions closely associated with depression, including the ventral tegmental area (VTA), nucleus accumbens (NAc), prefrontal cortex (PFC), hippocampus (hip), DRN, and LHB. In addition, this review discusses the potential limitations and future directions of these techniques, with the aim of providing reliable insights and guidance for their use in investigating the neural circuitry of depression.

## 2 Overview of optogenetics

**Figure 1:**
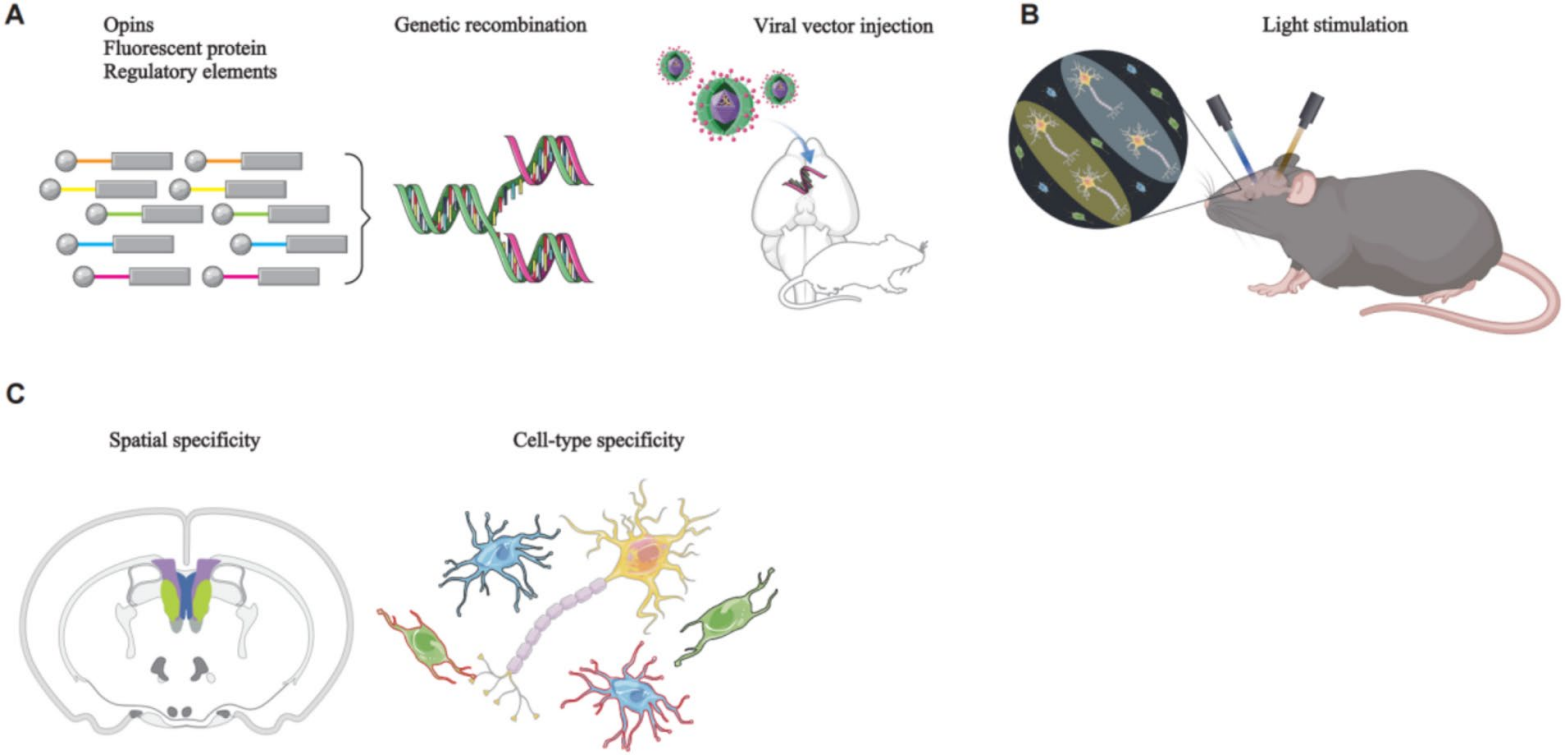
The principles of optogenetics manipulation of rodent neural circuits. **(A)** A genetic construct, comprising opsins, a fluorescent protein, and regulatory elements such as a promoter, is packaged into a viral vector. Stereotaxic surgery is then employed to deliver the viral vector to the target brain region. **(B)** Upon reaching the target brain region, the viral vector releases the genetic construct, specifically targeting the desired cell type. The expression of opsins modifies ion channel proteins within the target cells, rendering them responsive to specific wavelengths of light. Subsequent illumination of these cells with specific light wavelengths enables precise control of their activity. **(C)** Brain imaging techniques are used to visualize the specifically targeted cells.

Optogenetics is a cell-activity modulation technique that combines optical and genetics methods to modulate the activity of specific cell types (Deisseroth, 2010). It involves the use of light-sensitive proteins, also known as opsins, which are responsible for mediating light sensing in various organisms. These proteins convert light into intracellular signals, enabling organisms to respond to light stimuli (Zhang et al., 2011). Opsins are found in both prokaryotes and eukaryotes organisms, and the core mechanism of optogenetics involves the genetic modification and subsequent stimulation of ion channel proteins. Viral vectors are commonly used to deliver opsins to the targeted brain regions. Once expressed, these opsins modulate the activity of ion channels in target cells in response to specific wavelengths of light. This modulation of ion, which flow across the cell membrane, can cause membrane depolarization or hyperpolarization, thereby allowing precise control of cellular activity (Deisseroth, 2010) (Figure 1).

**Figure 2:**
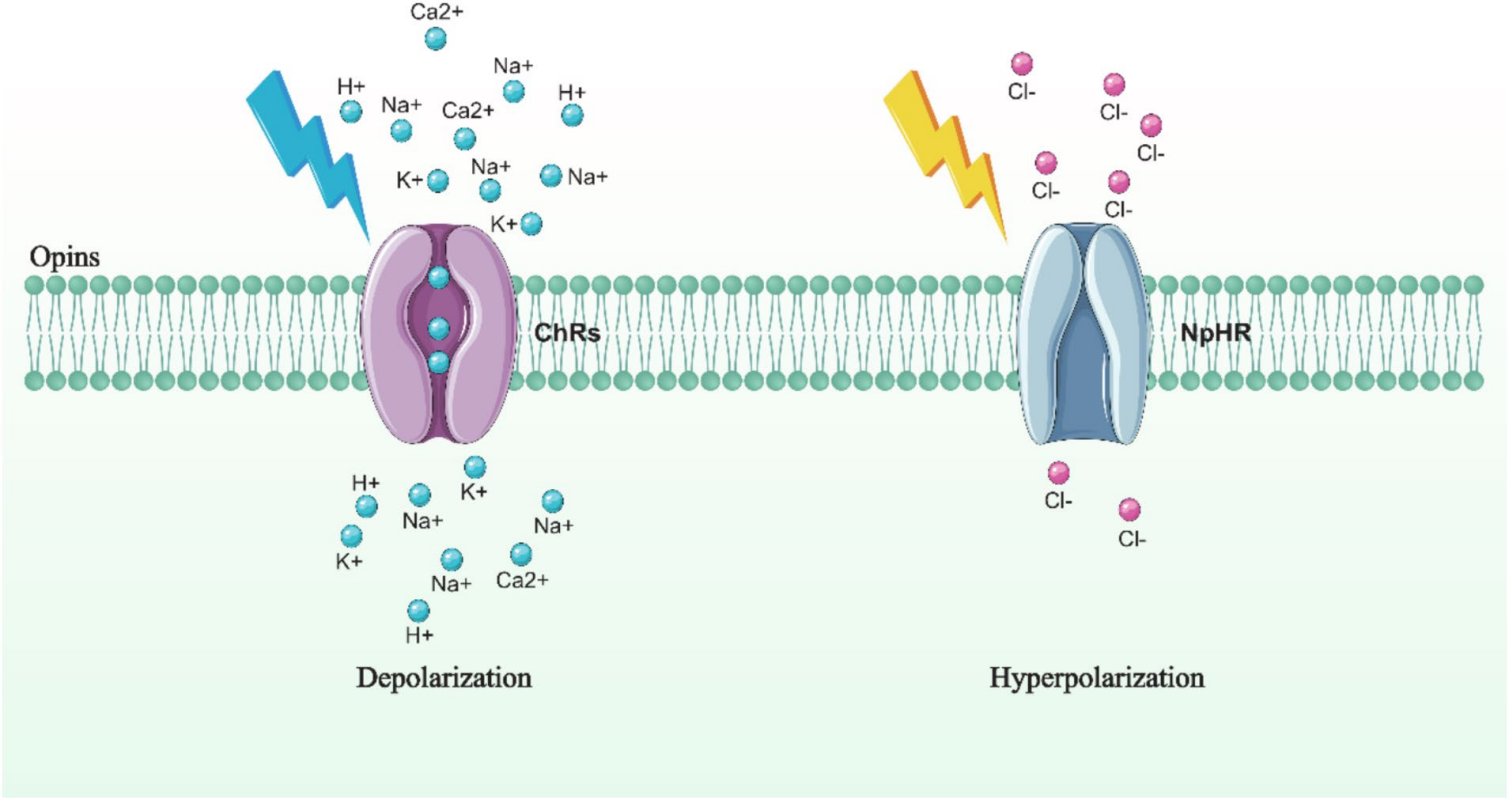
Principles of commonly used opsins. Illumination of ChRs embedded in the cell membrane with blue light of a specific wavelength induces a flow of cations across the membrane, resulting in cell depolarization (left). In contrast, illumination of NpHR on the cell membrane with yellow light of a specific wavelength causes the influx of anions from the extracellular space into the intracellular space, leading to neuronal hyperpolarization (right).

Opsins are primarily categorized into two types: excitatory and inhibitory. The most commonly used excitatory opsin in optogenetic research is the channelrhodopsin-2 (ChR2). ChR2 is a light-activated cation channel that permits the passage of sodium and potassium ions. When exposed to blue light with a wavelength of 460 nm, ChR2 opens, allowing the flow of cations influx across the membrane. This results in membrane depolarization and the subsequent activation of neurons. On the other hand, halorhodopsin (NpHR) is an inhibitory opsin, functioning as a light-activated chloride pump. When NpHR is illuminated with yellow light at a wavelength of 580 nm, it pumps chloride ions from the extracellular space into the intracellular space, resulting in membrane hyperpolarization and subsequent inhibition of neuron activities (Zhang et al., 2011) (Figure 2).

With advances in scientific technology, researchers have shifted their focus from seeking more effective opsins in nature to modifying existing opsins for enhanced optogenetics. Using techniques such as directed molecular evolution, scientists have introduced targeted mutations in ChR2, resulting in the development of a high-speed light-sensitive protein known as the engineered channelrhodopsin-2 variant (ChETA). ChETA generates larger photocurrents and exhibits faster kinetic changes, allowing for more rapid responses to and transmission of light signals (Gunaydin et al., 2010). Simultaneously, researchers have developed opsins activated by red light, known as red-shifted opsins. Red light, with its longer wavelength, has better tissue penetration, facilitating more effective modulation of deep brain regions and their target cells using optogenetics (Marshel et al., 2019). This advancement has enabled the exploration of biological mechanisms within these deeper brain regions. Furthermore, the recent development of dual-color opsins has allowed optogenetics to both activate and inhibit the same neurons within a single experiment. This is accomplished by illuminating dual-color opsins with different wavelengths of light, thereby permitting bidirectional control of neuronal activity (Vierock et al., 2021). Jaws is a halorhodopsin that is sensitive to red light, enabling deeper tissue penetration and enhancing the efficiency of optogenetic inhibition of neuronal activity in deep brain regions (Bansal et al., 2020). Guillardia theta anion channelrhodopsin (GtACR) is an anion channel sensitive to blue light. Unlike traditional ChRs, GtACR allows anions to flow into neurons upon light exposure, resulting in decreased neuronal activity. GtACR exhibits faster kinetics, leading to improved experimental results compared to ChRs, more rapid and pronounced changes in animal behavior, and more efficient neural modulation (Govorunova et al., 2015). A significant limitation of optogenetics stems from the transient nature of neuronal effects, which precludes the maintenance of long-term neuronal effects after a single light stimulus. Archaeorhodopsin-3 (Arch3) was developed to address this limitation. Arch3, a yellow-light-sensitive proton pump, mediates the efflux of protons from the cell upon activation, thereby inhibiting neuronal activity. It exhibits robust inhibitory efficacy and the capacity to generate more prolonged inhibitory effects (Chow et al., 2010). Complementary to the aforementioned bichromatic opsins that enable bidirectional modulation of neuronal activity within a single animal, SwiChR can also achieve activation or inhibition in the same cell. SwiChR is a bidirectional opsin that can function as either a cation or anion channel, with its specific modality of action determined by the intracellular chloride concentration (Rodriguez-Rozada et al., 2022).

## 3 Overview of chemogenetics

**Figure 3:**
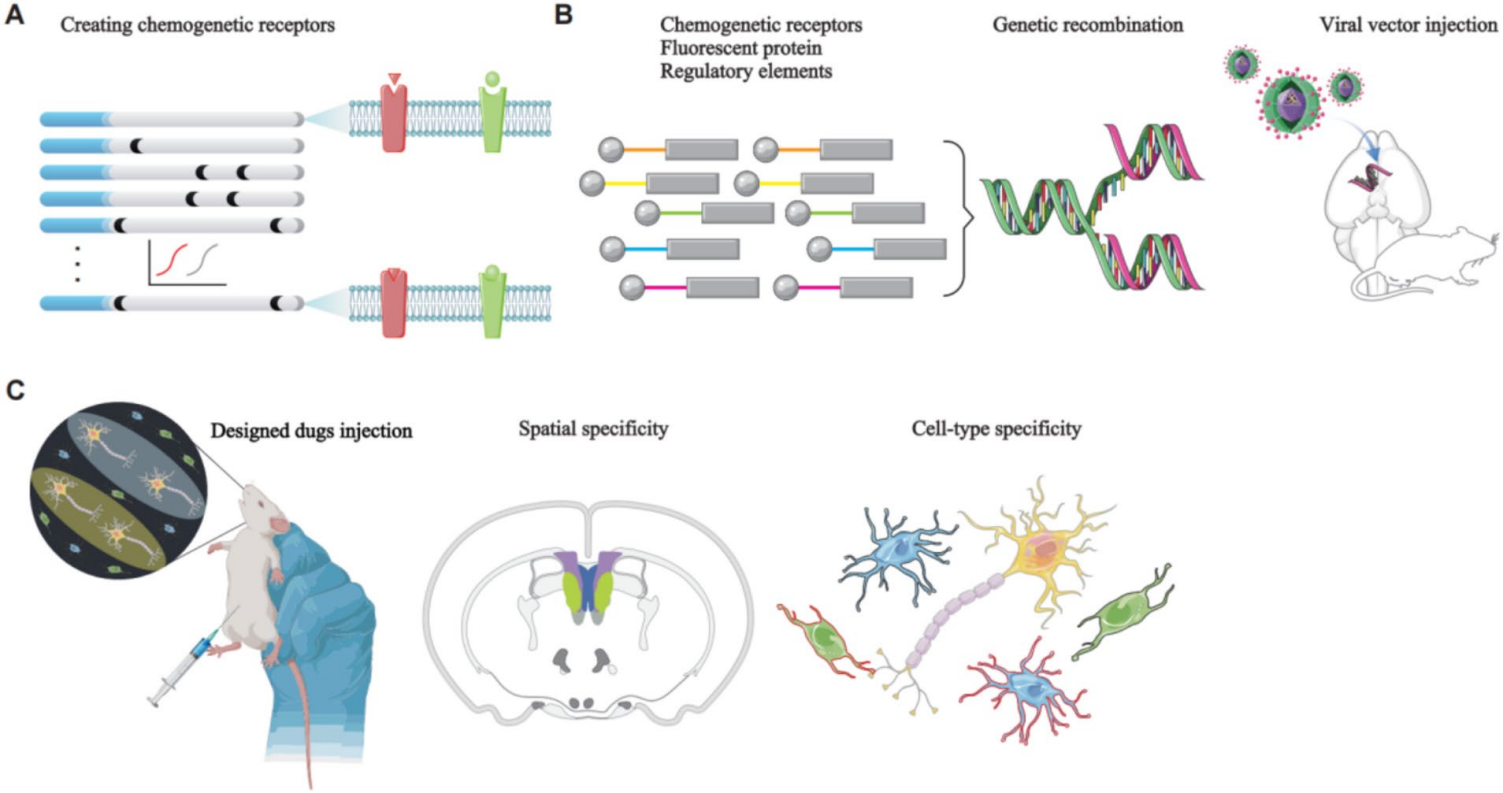
The principles of chemogenetics manipulation of rodent neural circuits. **(A)** Natural receptors are modified through directed molecular evolution to create chemogenetic receptors. **(B)** A genetic construct, consisting of a chemogenetic receptor, a fluorescent protein, and regulatory elements such as a promoter, is packaged into a viral vector. Stereotaxic surgery is then used to deliver the viral vector to the target brain region. The viral vector releases the genetic construct, which specifically targets the desired cell type, leading to the expression of chemogenetic receptors on the GPCRs of the target cells. These GPCRs are insensitive to endogenous ligands; however, they exhibit high sensitivity and affinity for specific designer drugs. Stimulation of these GPCRs with the designer drugs enables controlled modulation of target cell activity. **(C)** Brain imaging techniques are employed to visualize the specifically targeted cells.

Chemogenetics is a technique used in modulating cell activity by modifying receptor specificity and affinity, enabling the selective control of specific cell types (Roth, 2016). G protein-coupled receptors (GPCRs) constitute the largest known class of cell surface receptors play a crucial role in cellular signaling molecules. They exert diverse regulatory effects on cell activity, including activation and inhibition (Kawasawa et al., 2003). The core principle of chemogenetics involves the use of designer receptors activated exclusively by designer drugs (DREADDs), which rely on the modification and stimulation of GPCRs. This approach uses viral vectors to deliver chemogenetic receptors derived from natural receptors through mutagenesis into specific brain regions. These chemogenetic receptors, once expressed on target cells, are rendered insensitive to endogenous ligands but exhibit high sensitivity and affinity for specific designer drugs. Consequently, these designer drugs can be used to stimulate GPCRs, thereby enabling artificial control of target cell activity and function (Roth, 2016) (Figure 3).

**Figure 4:**
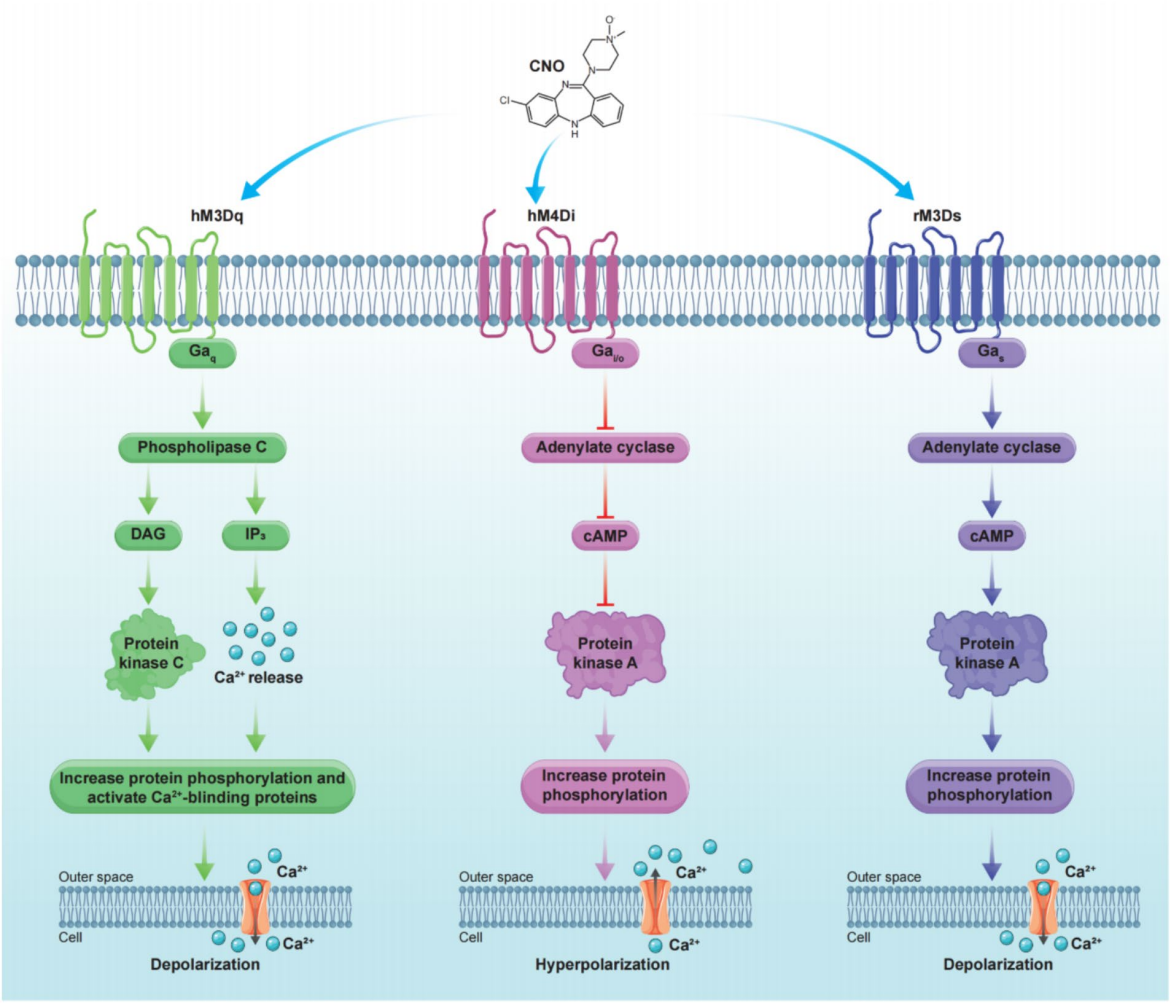
Principle of commonly used chemogenetic receptors. Stimulation of hM3Dq on the cell membrane with CNO initiates downstream signaling cascades that lead to cell depolarization (left). In contrast, stimulation of hM4Di on the cell membrane with CNO triggers downstream signaling cascades that result in cell hyperpolarization (middle). Stimulation of rM3Ds on the cell membrane with CNO triggers downstream signaling cascades that result in cell depolarization (right).

Researchers have employed directed molecular evolution to introduce site-directed mutations in human muscarinic receptors (hM), leading to the development of hMDREADDs, which are currently the most widely used chemogenetic receptors (Armbruster et al., 2007). hMDREADDs include three receptor types: Gq-coupled hM3DREADD (hM3Dq), Gi-coupled hM4DREADD (hM4Di), and Gs-coupled rM3DREADD (rM3Ds) (Galvan et al., 2019). These receptors leverage their respective G protein signaling pathways to modulate neuronal activity either by activating or inhibiting it. When expressed in neurons, hMDREADDs induce depolarization or hyperpolarization in response to clozapine-N-oxide (CNO), demonstrating their ability to modulate neuronal activity in a precise manner. Notably, in the absence of CNO, no change was observed in the resting membrane potential, indicating that hMDREADDs have negligible affinity for acetylcholine and are highly selective for CNO activation (Ma et al., 2022). This high selectivity for CNO activation facilitates the widespread application of hMDREADDs in research (Figure 4).

Beyond hMDREADDs, researchers have developed *κ*-opioid DREADDs (KORDs) through site-directed mutagenesis of κ-opioid receptors. KORDs exhibit minimal sensitivity to dynorphin but possess high affinity and sensitivity to salvinorin B (Vardy et al., 2015). The co-expression of KORDs and hM3Dq, which both modulate cellular activity through G protein signaling pathways, in the same neuronal population within a single animal allows for the bidirectional control of neuronal activity by activating each receptor with specific designer drugs.

## 4 Commonly used rodent models of depression and associated behavioral assays

Given the well-established connection between depression and stressful events, researchers often use acute or chronic stress paradigms to induce depression-like behaviors in animal models (Kendler et al., 1999; Kessler, 1997). Rodent models commonly employed for optogenetics and chemogenetics studies on depression include chronic social defeat stress (CSDS), chronic unpredictable mild stress (CUMS), and learned helplessness (LH) (Slattery and Cryan, 2017).

CSDS serves as a model to replicate human depression arising from various social stressors (Berton et al., 2006). Golden et al. (2011) established the standard CSDS paradigm, in which animals exhibit human-like depressive symptoms, including reduced social interaction, anhedonia, and abrupt changes in body weight. The primary advantage of CSDS is its ability to simulate the mechanisms underlying human depressive behavior at a social level, demonstrating robust structural validity and stability (Koolhaas et al., 1997). Compared to other non-social stress models, CSDS more closely approximates human conditions (Slattery and Cryan, 2014). Consequently, the CSDS paradigm is regarded as one of the most widely used models of depression (Bordes et al., 2023). However, in practical implementation, CSDS also presents certain limitations, such as a degree of symptomatic overlap with anxiety, which can mislead researchers. Furthermore, CSDS is primarily applicable to male rodents, as female rodents exhibit a deficiency in aggression and inconsistent attack behaviors during interactions with residents, limiting the study of depression primarily observed in females (Björkqvist, 2001; Shimamoto et al., 2015).

Preclinical studies demonstrate that uncontrollable, chronic stress impairs the brain’s reward circuitry (Guitart-Masip et al., 2023). Furthermore, repeated exposure to homogeneous stressors can induce adaptation in animal models, potentially resulting in a reduced manifestation of depressive- or anxiety-like behaviors (Parihar et al., 2011; Deng et al., 2017). Therefore, the CUMS model was established. This paradigm mitigates this effect by employing a series of stressors delivered in an unpredictable sequence, thus enhancing its suitability for investigating the neuropathological mechanisms and potential therapeutic targets of depression (Song and Kim, 2021). From a mechanistic perspective, the CUMS model is designed to recapitulate the progressive development of chronic depression, closely mirroring the experiences of depressed patients in response to stress (Antoniuk et al., 2019). The utility of the CUMS model is evidenced by its robust face and construct validity. The CUMS model induces persistent alterations in behavioral, neurochemical, neuroimmunological, and neuroendocrine parameters in animal models, closely resembling those observed in depressed patients compared to healthy controls. Consequently, the CUMS model is among the most extensively validated and widely utilized preclinical models of depression (Willner, 1997; Wiborg, 2013; Willner, 2005). The Wiborg (2013) paradigm is one of the most widely adopted and well-established CUMS models currently in use. However, the CUMS model also presents certain limitations in its practical application. For example, inter-laboratory variability in experimental conditions can influence the CUMS paradigm, leading to inconsistencies in model phenotypes. This variability may arise from factors including, but not limited to, the duration of the stress protocol, temperature, and humidity, potentially affecting the overall reproducibility and efficiency of the CUMS model (Willner, 1997).

The experience of helplessness is a cardinal symptom of depression and holds significant importance in both clinical and preclinical studies of this disorder. The LH model, one of the earliest animal models of depression, elicits depressive-like behaviors in model organisms through the application of unpredictable and uncontrollable foot shocks (Song and Kim, 2021; Seligman et al., 1968; Hitzemann, 2000; Seligman and Maier, 1967). Organisms subjected to the LH paradigm exhibit persistent low mood, weight loss, insomnia, and alterations in the activity of the hypothalamus-pituitary-adrenal (HPA) axis, closely mirroring the depressive symptomatology observed in humans clinically (Sesia et al., 2023; Cryan et al., 2005). The currently prevalent and widely employed LH paradigm is the one described by Vollmayr and Henn (2001). The primary strength of the LH model lies in the pronounced similarity between its manifestations and the clinical phenotype of severe depression, many aspects of which can be alleviated by antidepressant medications. It exhibits sound face, construct, and predictive validity. Moreover, the LH model has been instrumental in facilitating numerous investigations into depression, including studies examining its pathogenesis and evaluating novel therapeutic agents (Takamori et al., 2001; Pryce et al., 2011; Vollmayr and Gass, 2013). While this model has a substantial historical record, it is not without limitations. The principal drawback of the LH model is the transient nature of the depressive-like state observed in model organisms, which is characterized by an inability to sustain depressive-like behaviors over extended periods (Cryan et al., 2002). Furthermore, different strains of rodents exhibit variable sensitivity to LH, introducing an element of uncertainty into experimental designs (Henn and Vollmayr, 2005).

Beyond the three commonly used animal models of depression discussed earlier, there are other models that, while not extensively applied, are of notable importance to the field of depression research.

Pain, particularly neuropathic or trauma-induced pain, is a salient precipitant of depression (Li and Hu, 2016; Rosner et al., 2023). Research indicates that pain resulting from nerve damage can modulate depressive mood and induce neuronal death in brain regions associated with depression (Meerwijk et al., 2013). Furthermore, chronic pain and depression frequently present as comorbid conditions, and the mechanistic underpinnings of the pain-depression relationship are highly complex and remain incompletely elucidated (Micov et al., 2020; Doan et al., 2015; Harrass et al., 2021). Currently, pain-related models of depression are not considered mainstream. They primarily consist of models in which depressive-like behaviors have been observed in animals during pain investigations. Such models are generally characterized by a lack of stability and are still under active investigation. Examples include the complete Freund’s adjuvant-induced inflammation model, the carrageenan-induced hind paw edema model in rats, and models employing chemical stimuli (such as formalin, acetic acid, and capsaicin injection), which have demonstrated the presence of anhedonia and other depressive-like behaviors (Kaliyaperumal et al., 2020; Morado-Urbina et al., 2014; Winter et al., 1962; Stein et al., 1988). Although the mechanistic basis remains unclear, limiting the wider application of pain models for depression, the availability of these models provides a valuable resource for studies aimed at unraveling the underlying mechanisms.

Surgical models represent a relatively recent innovation in preclinical depression research, with bilateral olfactory bulbectomy (OBX) being the most commonly employed surgical procedure. In rodents, the olfactory system is a key regulator of behavior and emotion, exerting significant influence on endocrine, immune, and nervous system function (Hendriksen et al., 2015). Following OBX, model animals typically exhibit hyperactivity, diminished social interaction, increased nocturnal activity, and deficits in learning and memory, which are consistent with clinical manifestations of depression (Song and Leonard, 2005). Studies suggest that dysfunction within the cortical-hippocampal-amygdala circuitry underlies the depressive-like phenotype observed in these animals, implicating this network in the pathophysiology of depression (Price and Drevets, 2012). Moreover, antidepressant treatment can ameliorate depressive-like behaviors in OBX models. Despite demonstrating phenotypic stability and predictive validity, the OBX model has limitations in etiological and construct validity, which precludes its widespread adoption (Hendriksen et al., 2015).

Progress in genetic technologies has enabled the development of transgenic rodent models of depression. In recent years, numerous mouse strains have been generated and employed to investigate genes implicated in depression (Ridder et al., 2005; Urani et al., 2005; Pliakas et al., 2001). For instance, 5-HT^−/−^ mice exhibit a stable depressive-like phenotype, providing a valuable tool for exploring the underlying mechanisms of depression (Murphy and Lesch, 2008; Holmes et al., 2003; Renoir et al., 2008).

In summary, the development and application of rodent models of depression have significantly advanced research into the pathogenesis and neural circuitry of this condition. Each model possesses unique predictive, phenotypic, and construct validities, yet is also subject to inherent limitations, necessitating careful model selection by investigators.

Depression-like behaviors in experimental animals were assessed using a series of behavioral assays designed to capture the core symptoms of depression, such as anhedonia, social withdrawal, and loss of motivation. Common behavioral assays used in depression research to assess depression-like behaviors include the sucrose preference test (SPT), forced swim test (FST), tail suspension test (TST), and social interaction tests (SIT) The open field test (OFT), elevated plus maze test (EPM), and novelty suppressed feeding test (NSFT) are commonly used to assess anxiety-like behavior (Hao et al., 2019).

## 5 Applications of optogenetics and chemogenetics in the study of depression’s neural circuits

**Figure 5:**
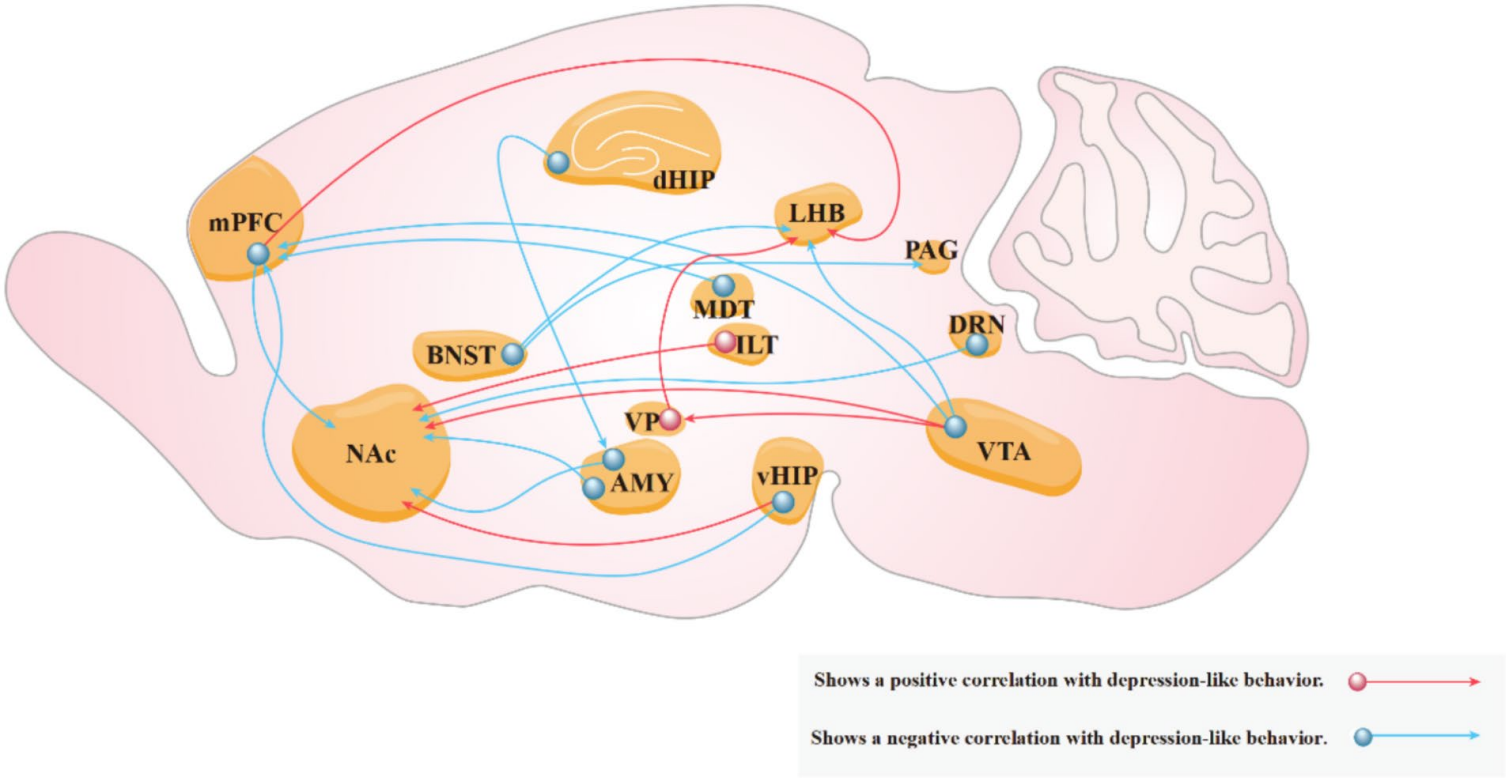
Neural circuits associated with depressive behaviors. Red arrows indicate that increased activity in the circuit exacerbates depression-like behaviors in experimental animals, while blue arrows indicate that increased activity in the circuit reduces these behaviors.

The advent of optogenetics and chemogenetics has significantly advanced our understanding of neural circuits in patients with depression. Compared with traditional pharmacological approaches, these techniques allow precise control over cellular activity, enabling researchers to target and manipulate the activity of specific neuronal populations. Their use in conjunction with various brain imaging and neurophysiological and behavioral assays has elucidated the relationship between various neural circuits and the pathogenesis of depression-like behaviors of rodents (Figure 5). Optogenetics is often preferred for its ability to control neural circuits compared to chemogenetics. However, optogenetics presents greater technical challenges and higher experimental costs than those of chemogenetics. Therefore, chemogenetics, which involves the use of engineered receptors activated by specific drugs remains a viable option for studies requiring long-term neural manipulation or when resources are limited. This review summarizes the progress in utilizing optogenetics and chemogenetics in neural circuit research of rodent models of depression, with a particular focus on brain regions implicated in depression.

### 5.1 Ventral tegmental area

Clinical studies have shown that dopamine (DA) expression and metabolism levels are significantly reduced in the brains of individuals with depression compared to healthy controls (Belujon and Grace, 2017). The mesolimbic reward pathway, mediated by DA, is a key contributor to depression. The VTA, which serves as an upstream region in this pathway, is closely associated with the pathogenesis of depression. Research has shown that in mice subjected to CSDS, altered activity of VTA DAergic neurons is crucial in the development of depression (Cao et al., 2010). In a notable study, Tye et al. (2012) employed optogenetics to inhibit VTA DAergic neurons in normal mice, resulting in a significant increase in immobility time in the FST and a significant decrease in the sucrose preference index in the SPT, thereby inducing depression-like behaviors. Subsequently, activating VTA DAergic neurons in mice subjected to CUMS using optogenetics led to significant improvement in depression-like behaviors This suggests a positive correlation between VTA DAergic neurons activities and depression-like behaviors in mice. In addition, these findings provide insights into the pathogenesis of depression. Notably, the study employed the rapid response capabilities of optogenetics to intermittently light-stimulate the model mice, observing distinct behavioral states. This approach enhances the reliability and rigor of the research findings. Furthermore, combining optogenetics with *in vivo* electrophysiology allowed researchers to observe the activity of targeted neurons in real-time, contributing to a more comprehensive understanding of the relationship between deep neural circuit mechanisms and depressive behaviors of rodents.

Transgenic mice lacking the kinase responsible for tyrosine hydroxylase phosphorylation in VTA DAergic neurons exhibit increased anxiety-like behaviors, a reduced sucrose preference index, and increased latency to eat in the NFST compared to wild-type mice. Zhong et al. (2014) employed chemogenetics to activate DAergic neurons in the VTA, which led to improvements in depression-like behavior in transgenic mice. Subsequent western blot (WB) analysis revealed an increase in DA release, which restored cyclic adenosine monophosphate (cAMP) signaling, thereby increasing tyrosine hydroxylase phosphorylation levels and improving the depression-like phenotype in these transgenic mice. The combined use of chemogenetics and WB blotting provides insights into the underlying biological mechanisms by which VTA-DAergic neurons mediate depression-like behaviors of rodents.

### 5.2 Nucleus accumbens

The NAc, a key region in the mesolimbic reward pathway, primarily receives projections from VTA-DAergic neurons (Fox and Lobo, 2019). The NAc is involved in regulating emotions such as pleasure and fear in mammals, as well as brain effects related to reward, addiction, and motivation (Chan et al., 2018; Zhu et al., 2023). It is also closely associated with depression. Deep brain stimulation of the NAc has been shown to significantly improve depressive symptoms (Song et al., 2024). Approximately 95% of the neurons within the NAc are medium spiny neurons (MSNs), which are primarily divided into those expressing DA D1 and D2 receptors (Drd1 and Drd2) (Chen et al., 2021). Francis et al. (2015) employed optogenetics to activate Drd1-MSNs in mice subjected to CSDS. They observed a significant increase in the SPT sucrose preference index and increased social interaction time in the SIT, thereby ameliorating depression-like behaviors in mice. Subsequently, inhibiting Drd1-MSNs in normal mice using optogenetics resulted in a significant reduction in both sucrose preference index and social interaction time in behavioral assays, thereby inducing depression-like behaviors. These findings indicate that Drd1-MSNs in the NAc can bidirectionally regulate depression-like behaviors in mice, highlighting the precise role of NAc in the pathogenesis of depression. Prior to the optogenetic manipulation of neurons, researchers employed a whole-cell patch-clamp to measure miniature excitatory postsynaptic currents (mEPSCs) in Drd1-MSNs. They found that the slope of mEPSCs in the Drd1-MSNs of mice following CSDS was significantly reduced compared to that in normal mice, suggesting that depression may lead to reduced Drd1-MSN activity. The combined use of optogenetics and whole-cell patch-clamp technique allows for precise targeting and clearer identification of target cells, thus enhancing the reliability of the experimental results.

Markovic et al. (2021) investigated the function of the VTA-NAc circuit using chemogenetics in a mouse model of inflammatory pain induced by complete Freund’s adjuvant injection into the paw. The authors observed a decrease in the sucrose preference index, indicative of anhedonia, a symptom of depression. Activation of VTA-NAc DAergic neurons using chemogenetics significantly increased the sucrose preference index, suggesting that targeting the VTA-NAc circuit may be a potential therapeutic approach for anhedonia, thus improving depression-like behaviors. Notably, prior to manipulating neurons using chemogenetics, researchers employed fiber photometry to record calcium signals from target cells in real-time, monitor cell activity, and ensure more precise targeting and reliable experimental results.

The olfactory tubercle (OT), along with the NAc, constitutes part of the ventral striatum and plays a role in reward, addiction, and motivation in mammals (Murata et al., 2019). It is closely associated with depressive behavior. Zhang et al. (2023) identified a close relationship between depressive behaviors and neurons expressing DA D3 receptors (Drd3) in the OT. Using optogenetics, Drd3 neurons were activated in mice that were successfully modeled using CRS. They observed a significant decrease in the immobility time in the TST and FST, thereby alleviating depression-like behaviors in these mice. These findings suggest a positive correlation between OT-Drd3 neuronal activity and depression-like behavior in mice. Subsequently, immunofluorescence staining revealed numerous positive neurons within the VTA-NAc circuit in the brains of these mice, suggesting that this circuit was also activated. This preliminary finding suggests that OT-Drd3 neurons may exert antidepressant effects by modulating the VTA-NAc circuit. These findings provide a foundation for further investigation into the mechanisms underlying the antidepressant effects of the VTA-NAc circuit. This study also suggests that the combined use of optogenetics and immunofluorescence staining may provide researchers with a valuable tool for exploring deeper regulatory relationships and clarifying the complex and ambiguous mechanisms of neural circuits.

### 5.3 Medial prefrontal cortex

Clinical studies have shown that patients with depression exhibit significant abnormalities in PFC function, particularly in the medial prefrontal cortex (mPFC) (Pizzagalli and Roberts, 2021). Deep brain stimulation of the PFC has been shown to significantly improve depressive symptoms in clinical settings, with optimal results observed when stimulating the mPFC (Widge et al., 2019). Collectively, these findings suggest that the mPFC is a potential target for the treatment of depression. The mPFC, as a core brain region of the mesolimbic reward pathway, receives projections from VTA dopaminergic neurons (Su and Si, 2022). Chaudhury et al. (2012) employed optogenetics to activate the VTA-mPFC DAergic neurons in a mouse model of CSDS. Behavioral analyses revealed a significant increase in both social interaction and sucrose preference indices, indicating an improvement in depression-like phenotypes in these mice. Subsequently, electrophysiological recordings demonstrated a significant reduction in the activity of VTA-mPFC DAergic neurons in mice exhibiting CSDS compared to control mice, further suggesting the involvement of this circuit in depression-like behaviors. The combined use of optogenetics and electrophysiological recordings has enriched research on the mechanisms of the mesolimbic reward pathway.

Glutamate, released by glutamatergic pyramidal neurons, and gamma-aminobutyric acid (GABA), released by GABAergic interneurons, are the two most prevalent excitatory and inhibitory neurotransmitters in the central nervous system. The balance between these two types of synaptic activity within neural circuits forms a microcircuit essential for maintaining normal brain function. When this balance is disrupted, it can lead to neural circuit dysfunction (McGrath et al., 2022). Fogaça et al. (2020) suggested that depression might result from abnormalities in this neural microcircuit in the mPFC. Specifically, they posited that excessive inhibition of glutamatergic pyramidal neurons by GABAergic interneurons within the mPFC, including their major subtypes, parvalbumin (PV)- and somatostatin (SST)-containing interneurons, contributes to depression. In addition, other researchers have employed chemogenetics to inhibit the activity of GABA, PV, and SST interneurons in several strains of transgenic mice (Gad1-cre, PV-cre, and Sst-cre mice) that have been successfully modeled using CUMS. The result revealed a significant reduction in immobility time in the FST and latency in the NSFT, indicating an improvement in depression-like behaviors and supporting the validity of the hypothesis. To further validate this hypothesis, researchers utilized whole -cell patch clamp to detect EPSCs and IPSCs in GABAergic interneurons and glutamatergic pyramidal neurons. The results revealed a significant decrease in the activity of GABAergic interneurons and a significant increase in the activity of glutamatergic pyramidal neurons, providing empirical evidence for the existence of this microcircuit.

Adenosine triphosphate (ATP), the primary energy source for cells, also serves as a crucial mediator of communication between neurons and astrocytes (Tan et al., 2017). Studies have shown an association between abnormal extracellular ATP levels in the mPFC neurons and depression (Xiong et al., 2019; He et al., 2021); however, the specific underlying mechanisms remain unclear. Lin et al. (2022) proposed that ATP exerts its antidepressant effects by inhibiting GABAergic interneurons within the mPFC-LHB circuit. To validate this hypothesis, researchers initially employed chemogenetics to activate GABAergic interneurons within the mPFC-LHB circuit in normal mice. In the SPT, the sucrose preference index of these mice decreased significantly, and their social interaction index decreased significantly in the SIT, indicating depression-like behaviors. Subsequently, researchers have employed chemogenetics to inhibit GABAergic interneurons within the mPFC-LHB circuit in mice after CSDS. This manipulation resulted in a significant increase in both the sucrose preference index and the social interaction index, thereby ameliorating the depression-like phenotype. This suggests that the mPFC-LHB circuit can exert a modulatory influence on depression-like behavior in mice. Finally, chemogenetics was used to inhibit GABAergic interneurons within the mPFC-LHB circuit of Itpr2^−/−^ mice, a strain characterized by a specific knockout of Itpr2, which results in reduced ATP levels and depression-like behaviors. These findings resulted in a significant increase in both the sucrose preference and social interaction indices, ameliorating depression-like behaviors and providing further evidence that ATP exerts antidepressant effects by inhibiting GABAergic interneurons within the mPFC-LHB circuit. Collectively, these findings underscore the potential of chemogenetics for investigating the mechanisms of endogenous cellular compounds in the pathogenesis of depression of rodents.

Ketamine, a noncompetitive N-methyl-d-aspartate receptor antagonist (Zanos and Gould, 2018), exhibits rapid antidepressant effects following a single administration in clinical settings (Jelen and Stone, 2021); however, the precise underlying pharmacological mechanisms remain elusive. Hare et al. (2019) proposed that the antidepressant mechanism of ketamine might involve an increase in mPFC-Drd1 neuronal activity. To investigate this hypothesis, researchers have employed optogenetics to activate mPFC Drd1 neurons in mice subjected to the FST. They observed a significant reduction in immobility time in the FST and a significant increase in the time spent in the open arms of the EPM, indicating a significant improvement in depression-like behavior in these mice. This antidepressant effect was both rapid and sustained, mirroring the effects of ketamine. Subsequently, researchers employed optogenetics to inhibit mPFC Drd1 neurons in mice following FST that had been administered ketamine administration. Behavioral assessments revealed a significant exacerbation of depression-like behaviors in these mouse models, attenuating the antidepressant effects of ketamine. These findings provide preliminary evidence supporting the proposed mechanism of action of ketamine, underscoring the potential of optogenetics for investigating both the pathogenesis of depression and the pharmacological mechanisms of antidepressants. Subsequently, researchers employed immunofluorescence staining to demonstrate that light stimulation of mPFC Drd1 neurons increased the number of positive cells in the basal lateral amygdala (BLA). These findings provide evidence for the existence of the mPFC-BLA circuit and suggest its potential role in regulating depressive behaviors of rodents, highlighting candidate neural circuits underlying the pathogenesis of depression for clinical research.

### 5.4 Hippocampus

Clinical magnetic resonance imaging (MRI) studies have revealed that the hip in individuals with depression is characterized by a significant reduction in volume (Roddy et al., 2019). Furthermore, electroencephalogram recordings have demonstrated a marked decrease in hip activity in individuals with depression, highlighting the hip as a critical brain region in depression research (Han et al., 2023). The dentate gyrus (DG), a subregion of the hippocampus, is implicated in depression (Tunc-Ozcan et al., 2019). Ramirez et al. (2015) employed optogenetics to activate DG neurons in mice subjected to the FST. This resulted in a significant increase in the SPT sucrose preference index and a significant increase in struggle time in the TST, indicating a marked improvement in depressive-like phenotypes. These findings suggest that activating DG neurons can positively regulate depression-like behaviors in mice, providing insights into the antidepressant mechanisms of the DG. Rawat et al. (2022) proposed that the rapid antidepressant effects of ketamine were mediated by the activation of adult-born immature granule neurons (ABINs) within the DG. To support this hypothesis, researchers have employed chemogenetics to inactivate DG-ABINs in mice that had been administered ketamine after CUMS. These mice exhibited a significant decrease in social and novelty indices in the SIT and a significant increase in immobility time in the TST. Subsequently, researchers employed chemogenetics to activate DG-ABINs in mice subjected to CUMS. No statistically significant differences in behavioral indices were observed between these mice and ketamine-treated mice following CUMS. These findings further clarify the pharmacological mechanism underlying the antidepressant effects of ketamine, demonstrating a positive correlation between DG-ABIN activity and depression-like behaviors in mice. This further underscores the potential of chemogenetics for investigating both the pathogenesis of depression of rodent and the pharmacological mechanisms of antidepressants.

Tyrosine kinase receptor B (TrkB), a transmembrane receptor protein, plays a critical role in the antidepressant activity (Duman et al., 2012). Carreno et al. (2015) demonstrated that TrkB exerts antidepressant effects by mediating the ventral hippocampus (vHIP)-mPFC circuit. Researchers have employed optogenetics to activate the vHIP-mPFC circuit in mice subjected to the FST. This resulted in a significant reduction in the immobility time during the FST, alleviating depression-like phenotypes in these mice. This finding demonstrates that activation of this circuit can produce antidepressant effects. To further elucidate the mechanism of action of TrkB, researchers pharmacologically activated TrkB in depressed mice, resulting in an improvement in depression-like behaviors. Subsequently, researchers employed optogenetics to inactivate the vHIP-mPFC circuit, leading to the reinstatement of depressive behaviors and the reversal of the antidepressant effects of TrkB activation. This confirms that TrkB exerts its antidepressant effects by modulating the activity of the vHIP-mPFC circuit. WB blotting was performed to assess TrkB expression levels in this study. The combined use of optogenetics and WB provides a valuable tool for investigating antidepressant neural circuit mechanisms involving specific receptors and functional proteins, paving the way for future advancements in this field.

### 5.5 Dorsal raphe nucleus

Clinical studies have revealed a significant reduction in the volume of the DRN in individuals with depression, highlighting the importance of the DRN in depression research (Matthews and Harrison, 2012). 5-Hydroxytryptamine (5-HT), a neurotransmitter that regulates mood, plays a critical role in depression, with brain 5-HT levels directly influencing the onset of this disorder (Sachs et al., 2015). The DRN contains a significant population of 5-HT neurons (Ikuta et al., 2017). Nishitani et al. (2018) employed optogenetics to activate DRN 5-HT neurons in mice during the TST. This resulted in a significant decrease in the immobility time, indicating an antidepressant effect. The antidepressant behavior ceased immediately after the light stimulus was ceased, suggesting that the activation of DRN 5-HT neurons directly produces antidepressant effects. To further investigate the specific antidepressant mechanisms of DRN 5-HT neurons, Nagai et al. (2023) employed optogenetics to activate DRN 5-HT neurons in mice subjected to CSDS. This resulted in a significant increase in social interaction time during social interaction tests and a significant decrease in immobility time in the TST, indicating a clear improvement in depression-like behaviors. Subsequently, immunofluorescence staining revealed a substantial number of positive cells in the VTA, colocalizing with specifically labeled DRN 5-HT neurons, providing evidence for the existence of a DRN-VTA circuit and its potential role in antidepressant mechanisms. To further validate these findings, the researchers employed optogenetics to activate the DRN-VTA circuit in CSDS mice, thus alleviating depression-like behaviors. Subsequently, they employed optogenetics to inhibit the DRN-VTA circuit in control mice, which induced depression-like behaviors. These findings further support the bidirectional regulation of depression-like behaviors by the DRN-VTA circuit.

Beyond behavioral and emotional changes closely linked to depression, loss of appetite and alterations in body weight are also recognized as clinical manifestations of the disorder (Harris et al., 1984). Animal models of depression often exhibit symptoms of decreased appetite and weight loss, providing insights into the physiological mechanisms of the disorder (Overstreet, 1991; Overstreet et al., 2013). Studies have established a critical role for melanocortin (MCR) in regulating food intake and metabolism (Liu et al., 2003). Mice with knockout MC4R genes and humans with MC4R gene mutations display hyperphagia and obesity, supporting the role of MC4R-expressing neurons in regulating appetite and obesity (Yen et al., 1994; Farooqi et al., 2003). However, the precise mechanisms underlying these behaviors and their relationship to depression remain elusive. Bruschetta et al. (2020) employed chemogenetics to inhibit MC4R neurons in the DRN of mice. This resulted in a significant reduction in appetite, an increase in immobility time in the FST, and a decrease in time spent in the center zone of the OFT, indicating the induction of depression-like behaviors. Conversely, activation of MC4R neurons in the DRN via chemogenetics led to an increase in appetite, a decrease in immobility time in the FST, and an increase in time spent in the center zone of the OFT, suggesting an antidepressant effect. These findings not only highlight the connection between appetite and depression but also clarify the specific mechanisms of MC4R action, providing a foundation for future investigations.

### 5.6 Lateral habenula

The LHb is considered the brain’s anti-reward center, regulating both the DAergic and 5-HTergic systems (Proulx et al., 2014). Various aversive stimuli activate it, and it is closely associated with depression (Nair et al., 2013; Huang et al., 2019; Yang et al., 2018). Clinical studies have shown that deep brain stimulation of the LHb can ameliorate depressive symptoms in patients, making it a widely recognized target for treating depression (Han et al., 2017). Sachs et al. (2015) employed chemogenetics to inhibit the neuronal activity of the LHb in mice subjected to CSDS. This intervention led to a significant decrease in immobility time during the FST and a significant increase in the social interaction index in the SIT, indicating an improvement in depression-like behaviors. In mice models resistant to the antidepressant effects of selective serotonin reuptake inhibitors (SSRIs), chemogenetic inhibition of LHb neuronal activity also resulted in a significant decrease in immobility time and a significant increase in the social interaction index, alleviating depression-like symptoms. These findings advance our understanding of the pathogenesis of depression and identify the specific targets of SSRIs, laying the foundation for the future development of first-line clinical drugs.

Zheng et al. (2022) discovered that the lateral hypothalamic (LH)-LHb circuit is closely associated with depression. The researchers employed chemogenetics to inhibit the LH-LHb circuit in mice subjected to CRS to elucidate the underlying mechanisms. This intervention led to a significant decrease in immobility time during the TST and a significant increase in sucrose preference index in the SPT, alleviating depression-like behaviors. To further identify the specific neuronal type mediating this circuit, the researchers used whole-cell patch-clamp recordings to assess neuronal activity in mice following CRS. This finding suggests that glutamatergic neurons mediate the LH-LHb circuit function. To further validate this finding, the researchers employed optogenetics to activate glutamatergic neurons within the LH-LHb circuit in mice. This significantly increased immobility time during the TST and decreased the sucrose preference index in the SPT, thereby inducing depression-like behaviors. For experimental rigor, the researchers also activated other neuronal types within the LH-LHb circuit using optogenetics; however, no significant behavioral differences were observed in these mice, further supporting the specific role of the LH-LHb circuit in depression. These findings highlight the significant advantages of optogenetics and chemogenetics in exploring the pathogenesis of depression at the level of individual neuronal types, thus greatly advancing the field of neural circuit research.

### 5.7 Other brain regions

Studies have revealed a significant reduction in volume, functional abnormalities, and molecular expression abnormalities in the entorhinal cortex (Ent) in both animal models of depression and individuals with depression, suggesting a close relationship between Ent and depression (Furtado et al., 2008; Kim et al., 2016; Chen et al., 2020; Uezato et al., 2009). Yun et al. (2018) employed chemogenetics to inhibit glutamatergic pyramidal neurons within the Ent-DG circuit in mice exhibiting depression-like behaviors. This intervention significantly increased latency during the NFST and increased social interaction time in the SIT, alleviating depression-like behaviors. These findings provide evidence of a relationship between the Ent-DG circuit and depression. Clinical studies have shown that repetitive transcranial magnetic stimulation (rTMS) of the visual cortex (Vis) can rapidly ameliorate depressive symptoms in patients (Zhang et al., 2020). However, research on the neural circuit mechanisms underlying these effects remains limited. Lu et al. (2022) discovered that the Ent Va-secondary visual cortex in the ipsilateral hemisphere (V2M) circuit was closely associated with depression. Researchers employed chemogenetics to inhibit the Ent Va-V2M circuit in mice to validate this hypothesis. These interventions resulted in a significant decrease in the social interaction index during the SIT, a significant decrease in the sucrose preference index during the SPT, and an increase in immobility time during the FST, inducing depression-like behaviors. Subsequently, researchers have injected retrograde viruses carrying different fluorescent proteins into various brain regions, including the V2M, PFC, NAc, BLA, and hip. Co-labeled neurons were observed in the Ent Va; however, the number of co-labeled neurons was minimal. These findings suggest that neurons mediating the Ent Va-V2M circuit constitute a distinct neuronal population. To further investigate this specific mechanism, researchers employed optogenetics to activate Va neurons in the V2M of mice subjected to CSDS. This intervention resulted in a significant increase in the social interaction index during the SIT, a significant increase in the sucrose preference index during the SPT, and a significant decrease in immobility time during the FST, alleviating depression-like behaviors. These findings support the bidirectional regulation of depression-like behaviors by the Ent-V2M circuit, suggesting the potential of V2M as a therapeutic target for depression and paving the way for further clinical research.

The substantia innominata (SI) is a subregion of the basal forebrain (BF) that extends from the BLA (Yetnikoff et al., 2015). Previous studies have indicated a strong association between SI and neuropsychiatric disorders (Cui et al., 2017; Heimer et al., 1996). In a study by Cui et al. (2022), optogenetics was used to activate glutamatergic pyramidal neurons in the SI of mice. Fiber photometry recordings revealed a significant increase in calcium signaling within glutamatergic pyramidal neurons in the LHb. Immunofluorescence staining further revealed a significant increase in the number of positive cells within the LHb, indicating activation of LHb glutamatergic neurons and confirming the existence of the SI-LHb circuit. To investigate the specific antidepressant mechanisms of this circuit, researchers have employed chemogenetics to activate glutamatergic pyramidal neurons within the SI-LHb circuit in mice. This activation resulted in a significant decrease in the sucrose preference index during the SPT, an increase in immobility time during the FST, and a decrease in time spent in the central zone during the OFT, all indicative depression-like behaviors. Subsequently, chemogenetics inhibition of glutamatergic pyramidal neurons within the SI-LHb circuit in mice subjected to CUMS alleviated depression-like behaviors. These findings highlight the potential of combining optogenetics and chemogenetics with other methods to explore the specific antidepressant mechanisms in neural circuits and to investigate neuronal projections within specific brain regions (Table 1).

**Table 1:** Summary of studies on depression examining optogenetic and chemogenetic approaches.

Region	Neuron’s type	Model	Neuromdoulation method	Primary findings	References
VTA	DA	CUMS	Optogenetic	Suggesting a positive correlation between VTA DAergic neuronal activity and depression-like behaviors in mice	Tye et al. (2012)
VTA	DA	TH^−/−^ mice	Chemogenetic	The antidepressant mechanism of VTA-DA neurons is characterized by an increase in VTA DA neuronal activity, leading to enhanced dopamine release. This, in turn, restores cAMP signaling, resulting in elevated levels of tyrosine hydroxylase phosphorylation and ultimately ameliorating depressive-like behaviors in mice	Zhong et al. (2014)
NAC	Drd1	CSDS	Optogenetic	Indicating that Drd1-MSNs in the NAc can bidirectionally regulate depression-like behaviors in mice	Francis et al. (2015)
VTA-NAC	DA	Pain	Chemogenetic	Activation of VTA-NAc DAergic neurons can improve depression-like behaviors in mice	Markovic et al. (2021)
OT	Drd3	CRS	Optogenetic	Suggesting that OT-Drd3 neurons may exert antidepressant effects by modulating the VTA-NAc circuit	Zhang et al. (2023)
VTA-mPFC	DA	CSDS	Optogenetic	Suggesting a positive correlation between VTA-mPFC DAergic neuronal activity and depression-like behaviors in mice	Chaudhury et al. (2012)
mPFC	GABA, pv, sst, Glu	CUMS	Chemogenetic	Suggesting that excessive inhibition of glutamatergic pyramidal neurons by GABAergic interneurons within the mPFC, including their major subtypes, parvalbumin (PV)- and somatostatin (SST)-containing interneurons, contributes to depression-like behaviors in mice	Fogaça et al. (2020)
mPFC-LHB	GABA	CSDS	chemogenetic	Suggesting that mPFC-LHB GABAergic interneurons can exert a modulatory influence on depression-like behavior in mice.	(Lin et al., 2022)
mPFC-LHB	GABA	Itpr2^−/−^ mice	Chemogenetic	Providing further evidence that ATP exerts antidepressant effects by inhibiting GABAergic interneurons within the mPFC-LHB circuit	Lin et al. (2022)
mPFC	Drd1	FST	Optogenetic	Suggesting that ketamine exerts its antidepressant action through the activation of Drd1 neurons in the mPFC	Hare et al. (2019)
mPFC-BLA	Drd1	FST	Optogenetic	Suggesting a positive correlation between mPFC-BLA Drd1 neuronal activity and depression-like behaviors in mice	Hare et al. (2019)
DG	ABINS	CUMS	Chemogenetic	Suggesting that the rapid antidepressant effects of ketamine are mediated by the activation of adult-born immature granule neurons (ABINs) within the DG	Rawat et al. (2022)
vHIP-mPFC	None	FST	Optogenetic	Suggesting that TrkB exerts its antidepressant effects by modulating the activity of the vHIP-mPFC circuit	Carreno et al. (2015)
DRN	5-HT	FST	Optogenetic	Suggesting that the activation of DRN 5-HT neurons directly produces antidepressant effects	Nishitani et al. (2018)
DRN-VTA	5-HT	CSDS	Optogenetic	Suggesting that DRN-VTA 5-HT neurons can bidirectionally regulate depression-like behaviors in mice	Nagai et al. (2023)
DRN	MC_4_R	MC4R^−/−^	Chemogenetic	Suggesting a positive correlation between DRN MC_4_R neuronal activity and depression-like behaviors in mice, highlighting the close association between depression and obesity	Bruschetta et al. (2020)
LHB	None	CSDS	Chemogenetic	Suggesting that the antidepressant mechanism of SSRIs involves the inhibition of LHB neuronal activity	Sachs et al. (2015)
LH-LHB	Glu	CRS	Chemogenetic, optogenetic	Suggesting an inverse relationship between the activity of LH-LHB glutamatergic neurons and depressive-like behaviors in mice	Zheng et al. (2022)
Ent-DG	Glu	CSDS	Chemogenetic	Suggesting an inverse relationship between the activity of Ent-DG glutamatergic neurons and depressive-like behaviors in mice	Yun et al. (2018)
Ent Va-V2M	None	CSDS	Optogenetic	These findings support the bidirectional regulation of depression-like behaviors by the Ent-V2M Va neurons	Lu et al. (2022)
SI-LHB	Glu	CUMS	Chemogenetic, optogenetic	Suggesting a positive correlation between SI-LHB glutamatergic neuronal activity and depression-like behaviors in mice	Cui et al. (2022)

In summary, the implementation of optogenetic and chemogenetic techniques in rodent models of depression has elucidated numerous relationships between neural circuit mechanisms and depressive-like behaviors. These neural circuits are likely intimately associated with the pathogenesis of human depression, offering a preliminary theoretical foundation for subsequent clinical investigations into the disorder’s pathophysiology. Furthermore, optogenetic and chemogenetic techniques can also serve as validation methodologies for antidepressants. These techniques allow for the exploration of antidepressant mechanisms, the investigation and validation of their efficacy in animal models, and provide a framework for clinical drug therapy and the development of novel therapeutics. Additionally, the delineation of neural circuitry underlying depressive behaviors in rodents can serve as potential targets for TMS. However, this requires further validation through primate studies and clinical trials. As discussed previously, the evolution of TMS is closely linked to advances in understanding depressive neural circuitry. Therefore, the application of optogenetics and chemogenetics represents an important catalyst for the evolution of TMS in the treatment of depression.

## 6 Discussion

Optogenetics and chemogenetics have emerged as powerful tools in neuroscience research; however, their applications are limited. This section discusses the key challenges associated with these techniques.

### 6.1 Opsins

A significant limitation of current optogenetics is the relatively low light sensitivity of commonly used opsins, which often necessitate the use of fiber-optic implants for light stimulation during experiments. This involves surgically implanting optical fibers into the brains of experimental mice, followed by light stimulation. Such invasive procedures can cause significant harm to animals, limit their free movement, and potentially introduce substantial errors into the experimental results. Recently developed opsins, such as ChRger2, have significantly improved light sensitivity, enabling researchers to induce neuronal excitation using red-light illumination without the need for implanted optical fibers (Bedbrook et al., 2019). This advancement significantly enhances the reliability of the experimental outcomes. Moreover, current optogenetics is primarily focused on controlling neuronal and neural circuit activity; however, they lack the precision to manipulate signal transmission along neuronal axons. To overcome this limitation, researchers have developed opsins ChR2-mGluR2-PA by incorporating a localization element, the metabotropic glutamate receptor 2-PA (mGluR2-PA), into ChR2 (Hamada et al., 2021). This novel protein specifically targets the glutamatergic neuronal axon terminals, enabling the induction of long-distance axonal synaptic transmission, thereby enhancing the precision of optogenetics. This development also points to a promising direction for future research in the development of light-sensitive proteins. Although chemogenetics can induce long-lasting effects on neuronal activity, optogenetics currently lacks the ability to achieve long-term manipulation of specific neuronal populations. Future research should focus on developing novel opinions to address this limitation. In summary, ongoing research continues to refine and expand the repertoire of opinions, advancing the field of optogenetics.

### 6.2 Designer drugs

CNO is the most widely used designer drug in chemogenetics. However, prolonged administration of CNO can lead to the desensitization of the target receptor, significantly reducing the effectiveness of the chemogenetic system. Recently developed designer drugs such as deschloroclozapine (DCZ) have shown promise in addressing this limitation. DCZ exhibits a high affinity and low desensitization probability in rodents and non-human primates, enabling long-term administration without compromising experimental quality (Nagai et al., 2020). However, further investigation is needed to understand its safety profile fully. CNO is metabolized into clozapine, a psychoactive drug that induces adverse effects upon administration (Tianme, 2022). These effects introduce uncertainty in the interpretation of the results, potentially confounding the identification of disease pathogenesis or drug mechanisms. Studies have shown that CNO can also increase anxiety-like behaviors in experimental animals, which is a limitation that currently lacks a definitive solution (Ilg et al., 2018). The novel designer drug JHU37160, a CNO analog, demonstrated significantly higher experimental efficacy than CNO (Bonaventura et al., 2019). It achieves comparable effects at much lower doses, thereby minimizing off-target effects and mitigating toxicity concerns associated with designer drugs. Unlike direct light stimulation in optogenetics, chemogenetic designer drugs require metabolic processing within the animal’s body to exert their effects, resulting in a slower response compared to optogenetics. This can be problematic for experiments requiring an immediate response. Future research should focus on developing novel designer drugs that address these limitations and improve the overall efficacy of chemogenetics.

### 6.3 Chemogenetic receptors

In addition to the commonly used chemogenetic receptors previously discussed, researchers have generated additional DREADDs through the mutagenesis of GPCRs (Nakajima and Wess, 2012). However, these newly created DREADDs often lack the desirable pharmacological properties of hMDREADDs, such as the specificity for certain designer drugs, which hinders their ability to precise control of targeted cell activity. Consequently, their widespread application is limited. Nevertheless, DREADDs can modulate cellular metabolism and enhance or inhibit the release of specific hormones (Wootten et al., 2018). This underscores the potential of mutagenesis and chimeric receptor engineering to expand the functionality of DREADDs, paving the way for novel avenues in chemogenetic research.

### 6.4 Viral vector

Viral transduction is the primary method used to deliver opsins and chemogenetic receptors. However, this method can potentially damage neurons, compromising the physiological functions of the experimental animals. In addition, traditional viral vector transduction methods often exhibit low efficiency, which may introduce uncertainty into the experimental outcomes. Recombinant adeno-associated viruses (rAAV) have been developed to address these limitations. The rAAV is a genetically engineered viral vector that offers several advantages over traditional vectors (Hudry and Vandenberghe, 2019). It exhibits low immunogenicity, causes minimal neuronal damage, and significantly enhances transduction efficiency (Tong et al., 2022). These benefits reduce experimental error and enhance the rigor of the experimental results. Transgenic Cre mice are frequently used for targeted delivery of drugs to specific neuronal populations. However, these mics are expensive and difficult to breed, presenting significant challenges for researchers. Specific viral vectors containing Cre recombinase have been developed to overcome these hurdles. These vectors can specifically target desired neurons within the brains of normal mice, simplifying the implementation of optogenetics and chemogenetics (Wang et al., 2021).

### 6.5 Associated techniques

Optogenetics and chemogenetics are often combined with *in vivo* electrophysiology to monitor the activity of targeted neurons in real-time. However, *in vivo* electrophysiology cannot selectively detect the activity of specific neuronal populations, which limits the precise manipulation of neural circuits and introduces uncertainty in research. The advent of fiber photometry, a technique that allows real-time monitoring of calcium signals in specific neuronal populations, significantly enhances the rigor of experiments by overcoming this limitation (Qi et al., 2022).

Fiber photometry is an established technique within the field of optogenetics, with fiber-optic calcium imaging being its most prevalent application. Calcium ions serve as essential intracellular signaling mediators in neurons. When neuronal excitation reaches the presynaptic terminal, voltage-gated calcium channels are activated, resulting in a substantial influx of calcium ions. This transient increase in intracellular calcium concentration triggers the fusion of synaptic vesicles with the presynaptic membrane, facilitating neurotransmitter release into the synaptic cleft. Subsequently, these neurotransmitters bind to postsynaptic receptors, propagating the signal to the downstream neuron and enabling sequential signal transmission. In resting neurons, intracellular calcium concentration typically ranges from 50 to 100 nM. During neuronal activity, this concentration increases by a factor of 10 to 100, providing a readily accessible means of monitoring neuronal activity. Fiber-optic calcium imaging is based on this principle (Zheng et al., 2015).

This technique relies on the precise correlation between intracellular calcium concentration and neuronal activity. Specialized fluorescent proteins, or genetically encoded calcium indicators (GECIs), transduce intracellular calcium concentration into a measurable fluorescence signal. This signal is then detected by a fiber optic recording system, enabling the monitoring of neuronal activity. GECIs, such as those incorporating green fluorescent protein (GFP), red fluorescent protein (RFP), and their variants, are commonly employed. These proteins bind to calmodulin (CaM) and the M13 domain of myosin light chain kinase. Increased neuronal activity triggers the opening of voltage-gated calcium channels, resulting in a substantial influx of calcium ions. These ions bind to CaM, inducing an interaction between the M13 and CaM domains, which leads to a conformational change in cpEGFP, thereby increasing the green fluorescence signal. In addition to GECIs, other specialized fluorescent probes have been developed, including those that are specific to neurotransmitters such as dopamine (DA), γ-aminobutyric acid (GABA), and glutamate (Falkner et al., 2016). These advancements in fluorescent probes enable the application of fiber photometry to investigate neuronal function and the neural circuitry underlying mental disorders, including depression. Further advancements in brain imaging technologies, such as *in vivo* patch-clamp recordings, two-photon calcium imaging, and MRI, are poised to expand the applications of optogenetics and chemogenetics.

Investigating the complex neurocircuitry involved in depression often requires manipulation of different brain regions and circuits across separate cohorts of animals, which increases both the cost and complexity of the research. Multichannel optogenetics, a technique that allows for the simultaneous stimulation of multiple brain regions and circuits through the implantation of multiple optical fibers in the same animal, effectively mitigates these challenges (Yu et al., 2020). This streamlined approach simplifies the operation of optogenetics and contributes significantly to the exploration of the intricate neural circuit mechanisms underlying depression.

The majority of studies employing optogenetics and chemogenetics have focused on rodent models, with relatively few studies involving primates, particularly in the context of depression. While prior studies have shown that optogenetic techniques can modulate neuronal activity in primates, the resultant effects on associated behaviors have been minimal and have failed to replicate the robust manipulation effects observed in rodent models (Han et al., 2009; Diester et al., 2011; Gerits et al., 2012; Jazayeri et al., 2012; Ohayon et al., 2013). A significant factor contributing to this disparity is the inability to deliver adequate light to targeted neuronal populations. This limitation is primarily attributable to the optical fibers typically employed in optogenetics. Restrictions in fiber diameter and light transmission efficiency prevent the delivery of a light source with sufficient intensity to effectively manipulate neuronal activity within the primate brain. Merely increasing the optical fiber diameter not only exacerbates the invasiveness for experimental animals and potentially leads to unstable experimental outcomes but also complicates the control of light delivery augmentation. This challenge has emerged as a substantial impediment to the broader application of optogenetic techniques in primate models.

However, with advancements in the field, several strategies have been proposed to mitigate this limitation. First, employing a sharpened optical fiber tip can increase the illumination cone without altering the fiber diameter. Nonetheless, this approach still encounters the fundamental issue of the optical fiber diameter (Acker et al., 2016; Dai et al., 2015; Sileo et al., 2018). While the illumination cone is expanded, the overall effect remains constrained by the diameter. Alternative solutions involve forgoing optical fibers and directly illuminating brain tissue. By using an artificial transparent dura mater to directly illuminate the cerebral cortex, the limitations imposed by the diameter of optical fibers are circumvented, thus enabling full light source provision (Ruiz et al., 2013; Chernov et al., 2018; Yazdan-Shahmorad et al., 2016). Regarding subcortical nuclei, red-shifted opsins may be utilized to enhance the depth of light penetration. However, considerable limitations persist (Chuong et al., 2014). Firstly, these approaches may lead to widespread infection in experimental animals, thereby confounding experimental outcomes. Secondly, deep brain regions remain challenging to access. The inherent scattering properties of light cause interference during its transit through tissue, diminishing manipulation efficacy. Long-term implantable illumination devices appear to offer a solution to these concerns. They enable prolonged delivery of a light source to targeted neuronal populations, mitigating the risk of infection associated with open chamber procedures and effectively addressing these challenges. However, limitations inherent to their use have also become evident, primarily the current incapacity of existing illumination devices to provide high-power, high-resolution illumination, which precludes the induction of acute neuronal responses (Yazdan-Shahmorad et al., 2015; Komatsu et al., 2017). To address this limitation, a long-term implantable light-emitting diode (LED) array has been engineered. This device allows for the manipulation of primate neuronal activity through high-throughput light delivery and has demonstrated the ability to effectively control related behaviors in rhesus macaques. This represents a relatively comprehensive optogenetic system for use in primates. However, some subtle deficiencies remain. For example, the significant heat generated by this device during light emission can potentially compromise the rigor of experimental findings. Furthermore, this device still cannot penetrate deep into brain nuclei, presenting challenges for further investigation of deep-seated biological mechanisms (Rajalingham et al., 2021).

Furthermore, this gap also is attributed to challenges associated with stereotaxic injections into primate brains, which are often larger and more complex. However, the emergence of stereotaxic instruments specifically designed for large animals, including fully automated stereotaxic systems, is expected to reduce these challenges significantly (Shen et al., 2023).

Although the current applications of optogenetics and chemogenetics in primates are limited and their efficacy has yet to be validated, active efforts are underway to advance research in these areas. Primates serve as a crucial translational bridge connecting preclinical studies to clinical treatments, and it is anticipated that a substantial body of research involving primates will emerge in the near future. This research is expected to facilitate the translation of findings from preclinical studies to clinical trials.

## 7 Emerging technologies: sonogenetics and odourgenetics

While optogenetics has advanced significantly, enabling non-invasive manipulation of targeted cells, its limitations, such as poor penetration depth and scattering of light in opaque tissues, can significantly reduce stimulation efficiency (Bashkatov et al., 2005). Sonogenetics has emerged as a potential solution for these challenges. Sonogenetics combines ultrasound with genetics to modulate cellular activity with high specificity (Ibsen et al., 2015). It leverages mechanosensitive channels, which are naturally occurring mechanoreceptors in the body that convert mechanical forces into intracellular signals, thereby enabling organisms to respond to mechanical stimuli (Otero-Sobrino et al., 2023). Ultrasound waves, with frequencies exceeding 20,000 Hz offer several advantageous properties, including safety, non-invasiveness, deep tissue penetration, and high spatial and temporal resolution (Zhang et al., 2023). These properties, along with their diverse biological effects, including thermal, cavitation, and mechanical effects, have led to the application of ultrasound in fields such as neurostimulation, tumor treatment, and gene and drug delivery (Darrow, 2019). The core principle of sonogenetics involves the use of viral vectors to express mechanosensitive channels within ion channels of target cells. Upon expression, ultrasound stimulation triggers ion flux across the cell membrane, resulting in membrane depolarization or hyperpolarization, thereby enabling the precise control of cellular activity (Ibsen et al., 2015). Due to its ultrasound-based approach, sonogenetics surpasses optogenetics in its ability to modulate deep brain nuclei and neural circuits (Xian et al., 2023). However, sonogenetics has limitations, including incompatibility with electrophysiology equipment, especially during low-frequency, long-duration stimulation, in which ultrasound vibrations can destabilize the electrodes and introduce experimental errors. Furthermore, the behavioral responses observed following direct ultrasound stimulation *in vivo* may be attributed to the activation of the auditory pathway via ultrasound vibrations within the inner ear, which poses a challenge to the effectiveness and mechanisms of sonogenetics (Sato et al., 2018). Notably, incorporating a deaf control group into experiments to enhance rigor adds complexity to the experimental design (Guo et al., 2018).

Chemogenetics offers advantages such as ease of use and prolonged biological effects compared to optogenetics; however, it exhibits a slow onset of action. Odourgenetics, a novel technique that utilizes odor stimuli to modulate cellular activity, addresses this limitation (Wu et al., 2024). Odourgenetics leverages odorant receptors (ORs), which are naturally occurring olfactory receptors that detect odors and translate them into intracellular signals, enabling organisms to respond to olfactory stimuli (Billesbølle et al., 2023; Zhao et al., 2024). The principle of odourgenetics involves using viral vectors to express ORs within ion channels of target cells. Once expressed, specific odorants trigger ion flux across the cell membrane, leading to membrane depolarization or hyperpolarization, thereby enabling the precise control of cellular activity. Odourgenetics surpasses optogenetics in sustaining target cell activity for prolonged periods and surpasses chemogenetics with its significantly faster onset of action, achieving efficacy within 5 min. However, odourgenetics has limitations, including difficulty in quantifying odor stimuli and potential interference from other odors during experiments, which can disrupt animal behavior and introduce experimental bias. While sonogenetics and odourgenetics have not yet been applied to the study of neural circuits involved in depression, their ongoing development holds immense promise for overcoming the limitations associated with optogenetics and chemogenetics, further advancing the investigation of neural circuit mechanisms underlying depression.

## 8 Conclusion

The advent of optogenetics and chemogenetics has enabled researchers to investigate the neurobiological mechanisms underlying depression at the neuronal level. These techniques allows for precise modulation of brain regions and neural circuits in animal models of depression, leading to either attenuation or exacerbation of depression-like behaviors. When combined with diverse neuroimmunological assays, behavioral analyses, and brain imaging techniques, these technologies have significantly advanced our understanding of the neural circuits in depression. This progress has led to the identification of novel therapeutic targets and diagnostic tools, which are crucial for the development of novel antidepressant drugs. Consequently, this advancement holds significant potential for improving clinical outcomes in the treatment of depression. Additionally, emerging techniques such as sonogenetics and odourgenetics, derived from optogenetics and chemogenetics, are being implemented in experimental settings. As these technologies continue to advance, they are expected to provide a clearer understanding of the neural circuitry underlying depression.
